# ColpoClassifier: A Hybrid Framework for Classification of the Cervigrams

**DOI:** 10.3390/diagnostics13061103

**Published:** 2023-03-14

**Authors:** Madhura Kalbhor, Swati Shinde

**Affiliations:** Department of Computer Engineering, Pimpri Chinchwad College of Engineering, Pune 411044, India; madhura.kalbhor@pccoepune.org

**Keywords:** colposcopy, feature extraction, machine learning, feature fusion, GLCM, GLRLM, HOG

## Abstract

Colposcopy plays a vital role in detecting cervical cancer. Artificial intelligence-based methods have been implemented in the literature for the classification of colposcopy images. However, there is a need for a more effective method that can accurately classify cervigrams. In this paper, ColpoClassifier, a hybrid framework for the classification of cervigrams, is proposed, which consists of feature extraction followed by classification. This paper uses a Gray-level co-occurrence matrix (GLCM), a Gray-level run length matrix (GLRLM), and a histogram of gradients (HOG) for feature extraction. These features are combined to form a feature fusion vector of the form GLCM + GLRLM + HOG. The different machine learning classifiers are used for classification by using individual feature vectors as well as feature fusion vectors. The dataset used in this paper is compiled by downloading images from the WHO website. Two variants of this dataset are created, Dataset-I contains images of the aceto-whitening effect, green filter, iodine application, and raw cervigram while Dataset-II only contains images of the aceto-whitening effect. This paper presents the classification performance on all kinds of images with the individual as well as hybrid feature fusion vector and concludes that hybrid feature fusion vectors on aceto-whitening images have given the best results.

## 1. Introduction

Cervical cancer affects the cervix of the vagina. Human Papillomavirus (HPV) is a major cause of cervical cancer. Other causes include smoking, sexually transmitted infections, and immune system dysfunction. Early detection of cervical cancer plays an important role in its prevention and treatment. Cervical cancer screening is necessary for early detection; however, less developed countries lack effective screening programs [1].

A Pap smear is the most common screening procedure that distinguishes abnormal cells and predicts the risk of cervical cancer. However, the Pap smear has limitations as incidences of both false negatives and false positives are high. If a Pap smear can predict the possibility of cancer with its limitations, then we suggest a colposcopy for a more accurate diagnosis. Colposcopy is a well-suited procedure for precancerous examination of the cervix that can also be used in a low-cost setting. The availability of professionals and technology plays an important role in the diagnosis and treatment of cervical cancer [1,2].

Colposcopy is a very subjective process as it depends on the knowledge and experience of the doctors. The primary aim of this test is to identify premalignant or malignant lesions as well as genital warts, polyps, and infections [3]. Visual inspection involves the application of acetic acid to the visible part of the cervix. 

The classification of a cervigram involves analyzing complicated patterns. Image analysis and machine learning methods are used widely in the medical field. These methods assist doctors by providing them with reasonable diagnoses. Primary features to detect abnormal cervix are aceto-whitening of the cervix, punctuations and mosaic patterns, erosion, and a rough surface [4].

Feature combination is already implemented in the literature related to colposcopy [5]. This paper uses statistical feature extraction methods such as Gray-level Correlation Matrix (GLCM) [6], Gray-level Run Length Matrix (GLRLM) [7], and Histogram of Gradients (HOG) [8]. GLCM is widely used in the medical field and is also used particularly in cervical lesion classification [9]. GLCM covers a wide set of texture features that are important to specifically analyze the cervix and considers pixels and their neighboring pixels. Features descriptors such as Harlick, Soh, and Clausi are considered to extract features. GLRLM is another a useful method for feature extraction in medical imaging and is also used in cervical lesion classification [5,7]. A histogram of gradients is also capable of recognizing texture patterns [8].

In this paper, features by using all three techniques, namely GLCM, GLRLM, and HOG, are extracted. Additionally, feature vectors obtained from GLCM, GLRLM, and HOG are combined to form a hybrid feature fusion vector. Machine learning algorithms, such as Naïve Bayes, Bayes Net [9], Random Tree, Random Forest [10], Decision Table, and Logistics, are used for classification [11,12,13,14]. 

Research papers in the literature have used their own dataset collected from some hospitals. No benchmark dataset is available online. However, WHO Atlas has provided a few images [3]. These images are of four types, namely Raw Cervigram, Aceto-whitening effect, Green Filter effect, and Iodine effect. Visual inspection of the cervix is conducted with the application of acetic acid and Lugol’s iodine on the surface of the cervix [15,16]. A cervigram is also viewed under a green filter to analyze mosaics and punctuations. We downloaded the individual images with their labels and performed image augmentation to increase the data size. As the size of these datasets is less, the handcrafted features are extracted, and image classification using machine learning is performed instead of deep learning methods [17,18]. 

The contributions of this paper are as follows:

- Proposes the framework consisting of the extraction of hybrid feature fusion vector followed by the classification of cervigrams;
- Experiments with each feature extraction method and proposes the hybrid fusion vector consisting of GLCM + GLRLM + HOG for more accurate classification than that of the individual;
- Builds the dataset by downloading individual images from the WHO website along with a label and augmenting them with different operations. This dataset is made available for other researchers;
- Classifies the cervigrams by using different machine learning classifiers;
- Evaluates the classification performance of these classifiers by using different performance measures.

The main motivation to choose a hybrid framework is that the resulting feature set can capture multiple aspects of the image, including its texture, shape, and edges [19]. This can improve the accuracy and robustness of cervigram image classification. The combination of GLCM, GLRLM, and HOG features in a hybrid framework is a common approach in image processing and computer vision applications. Furthermore, each of these feature extraction methods has its strengths and limitations, and a hybrid approach can overcome some of these limitations. GLCM is sensitive to the direction of texture features, while GLRLM is invariant to direction. HOG can capture edge information that may be missed by GLCM and GLRLM.

The organization of this paper is as follows. Section 2 covers the detailed literature review, followed by Section 3, which describes the proposed methodology. Then, Section 4 outlines the experimental results and discussions. 

## 2. Related Work

Classification of cervical cancer involves mainly two approaches. The first approach is to use various handcrafted feature extraction techniques such as GLCM, Gabor features, LBP, etc., and/or morphological features of the cervigrams [20]. These features are fed to machine learning classifiers for classification. Another approach is to use deep learning models for feature extraction as well as for classification. Many times, deep learning techniques yield higher accuracy, but they need a larger amount of data. Additionally, the deep learning methods lack the interpretability of the results, which is important in medical applications. As the size of the dataset is less, this paper uses the first approach for cervigram classification [21]. 

Various feature extraction methods have been used on the cervigram dataset. Acosta-Mesa et al. [22] performed aceto-white temporal classification by using KNN. They obtained an accuracy of 65%, 66%, and 67% with values of k as 1, 10, and 20, respectively. Ye Rang Park et al. (2021) [5] applied both machine learning and deep learning approaches for the classification of colposcopy images. Various feature extraction methods were applied, such as GLCM, GLRLM, and LOG, and features were used for classification with ML algorithms. They obtained an accuracy of 74%, 76%, 71%, and 91% for XGB, SVM, RF, and Resnet-50, respectively. Muhammad Thohir et al. (2020) [6] used GLCM for feature extraction. A support vector machine is used for the classification of colposcopy images. The authors obtained the best accuracy of 90% with the SVM classifier and GLCM. Mercy Asiedu et al. (2019) [7] extracted color and texture features. These features are used for the training of support vector machines. The proposed system obtained an accuracy of 81.3%, 78%, and 80% for CIN classification.

Various deep learning approaches have also been used in the literature for cervical lesion classification. Masakazu Sato et al. (2018) [4] applied deep learning to the colposcopy dataset. Patients were classified into three groups: severe dysplasia, carcinoma in situ, and invasive cancer. The authors applied L2 and L1 regularization, dropout, and data augmentation. The accuracy of the validation dataset was 50%. Liming Hu et al.(2019) [23] implemented deep learning and obtained an AUC of 0.91. Bum-Joo Cho et al. (2020) [3] used CNN with Inception and Resnet. Five different class models were constructed for different labeling.

In the current literature related to cervical cancer, GLCM is used for feature extraction. However, we extracted GLCM Harlick features and also performed feature fusion of GLCM, GLRLM, and HOG on the cervical colposcopy dataset.

In this study [24], the authors compare the classification performance of machine learning and deep learning for the diagnosis of cervical cancer. Using cervical images, the researcher has worked on the automatic classification capabilities of the ML algorithms XGB, SVM, and RF, as well as the DL algorithm Resnet-50, to determine which approach would be most beneficial for clinicians in making an accurate diagnosis. The use of multiple algorithms, including SVM, increases the accuracy of the classification model. The proposed approach has some drawbacks, such as the requirement for a size of the dataset of medical images for developing and testing the classification model. The AUC values for XGB, SVM, and RF using a 5-fold cross-validation were 0.82, 0.84, and 0.79, respectively, and Resnet-50 was 0.97. 

This study [25] develops a very deep network to use colposcopy images to detect cervical cancer. The proposed method uses a deep neural network architecture and various activation functions to improve the accuracy of a cervical cancer diagnosis. It uses the ResNet-designed network, which was modeled after the ResNet18 architecture. The performance of the ResNet is examined in this study using three different activation functions, and the advantages and disadvantages of each are discussed. As a result, using three different networks, three networks were produced. All networks were trained and evaluated on a dataset of cervical images. The results showed that the activation functions of the designed residual networks with leaky and parametric rectified linear units (Leaky-RELU and PRELU) have accuracy values of 90.2 and 100%, respectively.

A novel Cervical Net Deep Learning structure and feature fusion with Shuffle Net structural features is proposed by this study’s author [26] for computer-aided diagnosis of cervical cancer. Image acquisition, image enhancement, feature extraction, feature selection, feature fusion, and classification are the main procedures in our cervical cancer screening system. Five different machine learning (ML) algorithms are used in this scenario to process the features. The feature fusion and deep neural network architecture allowed for highly accurate classification of cervical cancer. The effectiveness of the suggested method may be influenced by the size and caliber of the training dataset. In the SVM classifier, this system achieved the highest accuracy for 5 classes at 99.1% by combining the 544 most crucial features from the novel Cervical Net with the 544 from Shuffle Net. 

The Kernel Extreme Learning Machine (KELM) method, which is a development of the ELM method by including a kernel in the system, is used to classify pixel neighbor information using colposcopy images according to this study [27]. The use of GLCM for texture feature extraction and KELM for classification allowed for extremely accurate detection of cervical cancer. The dataset used in this study might only have a small sample size, which could lower the model’s precision. The results showed that a Gaussian kernel with the best neighborhood angle of 45 degrees had the highest accuracy, followed by a linear kernel with 78.5% accuracy and a polynomial kernel with 87.5% accuracy. When the GLCM is read using diagonal pixel readings, the probability of the data having a Gaussian distribution is increased.

The detection of cervical cancer from Pap smear images has been studied in this paper [28] using a Pap smear analysis tool. This study describes the development of a tool that uses Pap smear images to automatically identify and classify cervical cancer. A dataset of Pap smear images was used by the authors to train and test the PAT tool. The analysis of Pap smear images is automated by the PAT tool, which can speed up the procedure and reduce the chance of human error. The method performs better than many of the current algorithms when used on the Herlev benchmark Pap smear dataset, according to the results, with values of 99.28%, 97.47%, and 98.88%, respectively.

The authors of this paper [29] suggest a method for using Pap smear images for digital image processing in computer-assisted cervical cancer screening. The cervical cancer screening procedure consists of six basic steps. Segmenting cells, extracting features, selecting features, and classifying features are just a few of the steps involved in creating an image. Using the Herlev and SIPaKMed datasets separately, the average classification accuracy for the 2-class problem was 98.47%, and for the multi-class problem, it was 90.84% (7 classes) and 94.09% (5 classes). The proposed method improves the ability to distinguish between abnormal and normal cells, which is its main advantage.

The design of a thorough ensemble deep learning model for the automatic diagnosis of the WSI is covered in this paper [30]. The proposed network accurately discriminates between 4 classes up to 99.6% of the time. The use of precision and the speed of this work set it apart from previous studies. It concentrates not on a single cell but the entire stained slice image. The cervical cells with and without overlap are taken into consideration by the deep learning model that was created. The ensemble deep learning approach can improve the accuracy of cervical cancer screening. The proposed method has not been extensively tested on a large sample size and may require further validation studies.

To determine the class for the cervical cytology cell in the Pap smear image, a novel approach combining individual feature extraction with a classification technique is developed in this paper [31]. Feature extraction and classification are two of this paper’s main contributions. To evaluate the combined performance of individual methods in feature extraction, we have used individual features such as ERSTCM and EMSD texture features as one feature. Multiple kernels are combined to create a hybrid kernel SVM classifier that uses fuzzy logic to improve the classification. A proposed approach is compared with previously published works in individual feature extraction with the classification method; the proposed EMSD + FL-HKSVM produces a better result than the ERS TCM + FL-HKSVM, and the proposed CFE + FL-HKSVM produces a better result when combining individual feature extraction with the classification method in terms of the statistical parameters of sensitivity, specificity, and accuracy.

In this review [32], we have discussed cutting-edge methods that have been published in reputable works on computer-aided diagnostic systems for cancer detection. This review highlighted the methods examined and provided information to evaluate the methodology employed in the literature. This study offers recommendations for the creation of an automated, cost-effective disease classification system, which should be a big help to nations with scarce resources and treatment options. We also used different methods and techniques for the analysis of reviewing various literature. Experts and clinicians can identify effective algorithms and develop them for routine use in the diagnostic process due to the enormous potential benefits of computerized solutions for malignancy detection.

## 3. ColpoClassifier: Proposed Hybrid Framework for Classification of Colposcopy Images

**Figure 1:**
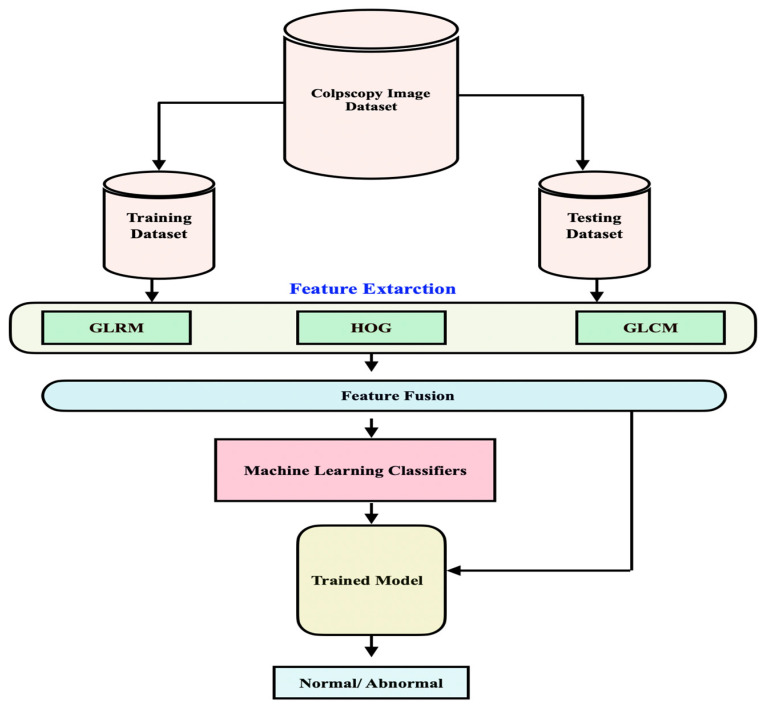
ColpoClassifier: proposed hybrid framework for classification of cervigrams.

The proposed framework is depicted in Figure 1. Colposcopy images are taken as input, and feature vectors from GLRLM, HOG, and GLCM are extracted and then combined to form a hybrid feature vector. Various machine learning classification algorithms are applied to hybrid feature vectors for classification.

### 3.1. Gray-Level Run Length Matrix (GLRLM)

GLRLM is a method for extracting texture features from images. In GLRLM, RGB channels for each colposcopy image are extracted, and features mentioned in Table 1 are extracted from each channel. These feature vectors are used for classification by machine learning classifiers. Seven GLCM features are extracted for each channel, as shown in Figure 1. Thus, in total, 21 features are extracted for an image. 

In GLRLM, the number of pairs of Gray-level values and their run lengths are considered [7]. A run is a group of pixels having the same value, which is consecutive. GLRLM is a histogram that records all combinations of intensities of gray values and runs for all specific directions. The value (*i*, *j*) in the matrix __ where *i* is a combination of Gray-level values and *j* is run length. There are four directions: horizontal (0 degrees), anti-diagonal (45 deg), vertical (90 deg), and diagonal (135 deg).

$$N=\sum_{i=1}^{N_{g}}\;\sum_{j=1}^{N_{r}}\;JP_{i,j}$$

From the above equation, *P* is used to denote GLRLM, then $P_{i,j}$ is (*i*, *j*) entry. *Nr* is a set of run lengths, while *Ng* is a set of different Gray levels, and N is the total number of pixels.

### 3.2. Gray-Level Co-Occurrence Matrix (GLCM)

GLCM contains information about the position of pixels having similar Gray-levels. Feature extraction from GLCM constitutes to features from Harlick [33], Soh [34], and Clausi [35]. Key features of GLCM known as Harlick features are listed in Table 2. Other features are also extracted. Similar to GLRLM, for each image, RGB channels are extracted, and then, for each channel, 22 GLCM features are extracted. So, for each image, 66 features are considered for classification.

The basic idea behind GLCM is texture, and it can be categorized as randomized patterns [36]. GLCM uses a co-occurrence matrix. The spatial relationship between the pixel and its neighboring pixel is considered. Joint distributions of pairs among images are calculated. The calculation of GLCM is computationally complex. Rows and columns in the co-occurrence matrix depend on the Gray levels. Element p(m,n) in the matrix represents occurrences of transitions between m and n. Relations among pixels are defined first, and then occurrences are calculated. Seven major feature descriptors of GLCM, known as Harlick descriptors, are shown in Table 2. Other feature descriptors are also used for feature extraction.

### 3.3. Histogram of Gradients (HOG)

HOG features measure the average gradient in the cell of an image concerning direction [8]. In this, for each image, 26,112 features are extracted, and PCA with 20 components is applied for feature reduction.

The main idea behind HOG is local object or shape in the image can be described by the distributions of intensity gradients and edge directions. The image is divided into small connected regions known as cells, and for pixels within cells, the histogram is compiled. The image gradient vector captures the changes in the magnitude of pixel colors. Steps for the calculation of HOG are shown as follows:

(a) Pre-processing.

An image is cropped to size 100 × 200 and resized to 64 × 128.

(b) Image gradients calculation.

To calculate the gradient of the image, both directions, that are horizontal direction and vertical direction, are considered, i.e., the image derivative is calculated from both directions. The magnitude and direction of the gradient can be calculated as

(1) $$g=\sqrt{gx^{2}+gy^{2}}\;\;\;\;\;\;\;\;\;\;\;\;\theta =arctan\frac{gy}{gx}$$

where g is the magnitude of gradient and θ is the direction of the gradient.

(c) Histogram of gradients.

The image is divided into cells of 8 × 8. For each cell gradient magnitude and gradient, the direction is calculated. Histograms of magnitude and direction are made and used for the classification.

### 3.4. Feature Fusion 

The features extracted by GLRLM, GLCM, and HOG are combined to form a hybrid fusion vector. The size of this hybrid fusion vector is 107, out of which 21 are of the GLRLM, 66 are the GLCM features, and 20 are of HOG.

### 3.5. Classification 

The hybrid feature fusion vector is given as input to different machine learning algorithms. Machine learning algorithms, such as Naïve Bayes, Bayes Net, Random Tree, Random Forest, Decision Table, and Logistic Regression, are used to classify cervigrams into the classes of normal or abnormal [9,10]. 

#### 3.5.1. Naïve Bayes

The Naïve Bayes algorithm is derived from Bayes’ theorem. Each feature pair is ranked independently of each other. The two assumptions of the characteristics of Naïve Bayes are independent, and all the characteristics are treated in the same way. The three types of Naïve Bayes classifiers: Gaussian, Bernoulli, and Multinomial. Gaussian Naïve Bayes is used for continuous data; Bernoulli Naïve Bayes is used for binary data; and Polynomial Naïve Bayes is used for count data [9,10,11]. 

#### 3.5.2. Bayes Net

Bayes nets are also known as Bayesian networks. Bayes net is based on a directed graph and does computation by using the chain rule. Probability distributions can be represented by directed acyclic graphs (DAG). DAG consists of vertices and edges, and vertices are connected by edges, which are one directional such that a closed loop is not formed [9,10,11].

#### 3.5.3. Random Tree

The probability distribution used to select the parent node, the expected number of nodes and edges in the tree, and the computational complexity of the algorithm used to build the tree. The properties of a random tree are the degree distribution, clustering coefficient, and diameter [9,10].

#### 3.5.4. Random Forest

A random forest is a supervised learning algorithm. A forest is a collection of decision trees and a supervised algorithm. The basic logic of the random forest algorithm is the decision tree. Decision trees essentially learn a hierarchy of if–then–else type problems that ultimately lead to classification. A decision tree asks the top node a series of questions with the highest priority. Then, several decision trees are run from the forest, and a majority vote is to give the final classification decision [9,10,12].

#### 3.5.5. Decision Table

Decision tables specify actions to take based on given conditions. This is usually expressed in the form of if–then rules. Formal representation of a decision process uses mathematical notation or logic, a method for generating a decision table or extracting decision rules from it, and the computational complexity to apply the decision table to new inputs [9,10,13].

#### 3.5.6. Logistics

In logistic regression, the probabilities are modeled by discrete outcomes given the input variables. It uses the sigmoid function to predict the class based on the aggregated input values. Logistic regression is a supervised ML algorithm; it predicts the probability of an output variable. The output of the target variable varies between 0 and 1 [9,10,14].

## 4. Dataset

**Figure 2:**
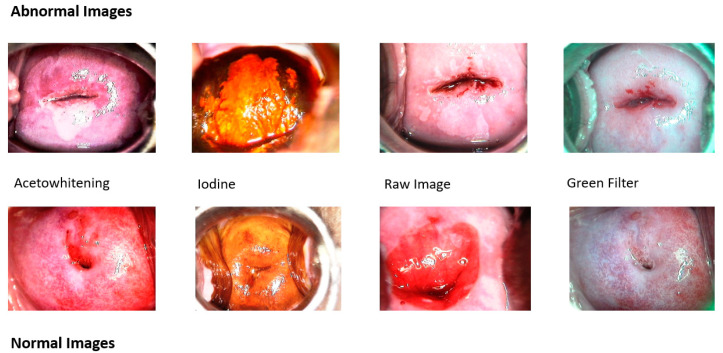
Sample images in the dataset for normal and abnormal classes—both classes have images of aceto-whitening, iodine, raw image, and green filter.

The dataset used in this paper is collected from patients’ cases in Atlas of Colposcopy: Principles and Practice of International Agency for Research (WHO) on Cancer [3]. Case studies include high-grade, low-grade, and normal colposcopy images. For each patient, there are images of the aceto-white effect, green filter, and Lugol’s iodine effect. High-grade and normal colposcopy images are considered in the dataset. Two sets of this dataset are formed for the experimentation, namely Dataset-I and Dataset-II. In Dataset-I, all kinds of given images are considered, i.e., images of aceto-whitening, green filter, and Lugol’s iodine, as well as raw images of cervigram, while in Dataset-II, only aceto-whitening images are considered. Some sample images of abnormal and normal classes are shown in Figure 2.

### Data Augmentation

As the data source contains very few images of each category, data augmentation is performed on both datasets to increase the number of images for experimentation.

Table 3 summarizes the different augmentation operations and corresponding parameter values.

After the augmentation with the above parameters, the resulting dataset has the size given in Table 4 and Table 5. Table 4 depicts the details of Dataset-I, having 370 total images, while Table 5 gives the details of Dataset-II, with 380 total images.

## 5. Experiments and Results

### 5.1. Implementation Details

Windows 10 operating system with an i3 processor is used for all computations. MATLAB and Google Collaboratory are used for feature extraction and image augmentation, respectively. In Google Colab, python libraries such as TensorFlow, Keras, and OpenCV are used. The freely available Weka tool is used for machine learning classification.

### 5.2. Performance Measures

To evaluate the performance of the proposed ColpoClassifier, the different performance measures, namely accuracy, sensitivity, specificity, precision, recall, and mean absolute error, are used. These are the widely used measures, specifically in medical applications. These are briefly defined as

- Accuracy (A) is the ratio of the number of correct predictions to all the predictions by the model and is given by
  (2) $$\mathrm{Accuracy}=\frac{\mathrm{TP}\;+\;\mathrm{TN}}{\mathrm{TP}\;+\;\mathrm{TN}\;+\;\mathrm{FP}\;+\mathrm{FN}}$$
  where TP is True Positive, TN is True Negative, FP is a False Positive, and FN is False Negative.

b. Sensitivity (True Positive Rate) is the proportion of correct predictions of the positive class. It signifies the classifier’s ability to accurately predict disease-positive patients and is given by
  (3) $$\mathrm{Sensitivity}=\frac{\mathrm{TP}\;}{\mathrm{TP}\;+\;\mathrm{FN}}$$

c. Specificity (True Negative Rate) is the proportion of negative predictions to the total negative cases. It signifies the classifier’s ability to distinguish the disease-negative patients and is given by
  (4) $$\mathrm{Specificity}=\frac{\mathrm{TN}\;}{\mathrm{TN}\;+\;\mathrm{FP}}$$

d. Precision (P) is the number of true positives divided by the total number of positive predictions.
  (5) $$\mathrm{Precision}=\frac{\mathrm{TP}}{\mathrm{TP}\;+\;\mathrm{FP}}$$

e. Recall (R) is the ratio of correctly classified positives to the total number of positives.
  (6) $$\mathrm{Recall}=\frac{\mathrm{TP}}{\mathrm{TP}\;+\;\mathrm{FN}}$$

f. Mean absolute error (MAE) is the error between expressions for the same event.
  (7) $$\mathrm{Mean}\;\mathrm{Absolute}\;\mathrm{Error}=\frac{\sum_{i=1}^{n}\left|yi-xi\right|}{n}$$

g. F1 measure (F1) is a weighted average of Precision and Recall.
  (8) $$F1\;\mathrm{Measure}=2\times \frac{P\times R}{P+R}$$

### 5.3. Results and Discussions

This section describes the experimental results of each feature extraction model with classification by different machine learning algorithms. This is followed by the results of a hybrid feature fusion vector with the same classifiers. These results are given for Dataset-I, followed by Dataset-II. For all the experimentation, 10-fold cross-validation is used to evaluate the robustness of the model.

#### 5.3.1. Dataset-I Results

- *GLRLM Feature Extraction for Dataset-I*

Table 6 depicts the experimental results of different classifiers with GLRLM features extracted from Dataset-I. It can be seen that the random forest has given a commendable accuracy of 68.11% as compared with other classifiers. Additionally, the values of sensitivity, specificity, precision, and recall are higher for the random forest classifier as compared to other classifiers.

b. *GLCM Feature Extraction for Dataset-I*

Table 7 summarizes the results of the different classifiers by using the GLCM feature extraction. Here, again, the random forest has given better accuracy of 65.94% compared with other classifiers. This accuracy and other values are lesser than the GLRLM technique of feature extraction.

c. *HOG Feature Extraction for Dataset-I*

Table 8 depicts the performance values of different classifiers based on the HOG feature extraction technique. The random forest classifier has given the highest accuracy of 69.72%. The HOG feature extraction method has given the highest performance values compared to GLRLM and GLCM feature extraction techniques.

d. *Hybrid Feature Fusion for Dataset-I*

Table 9 summarizes the performances of different classifiers trained on the hybrid feature fusion vectors by combining GLCM, GLRLM, and HOG. Here, again, the random forest has given a good accuracy of 72.43% compared to other classifiers. This accuracy is the highest compared to individual feature extractors.

**Figure 3:**
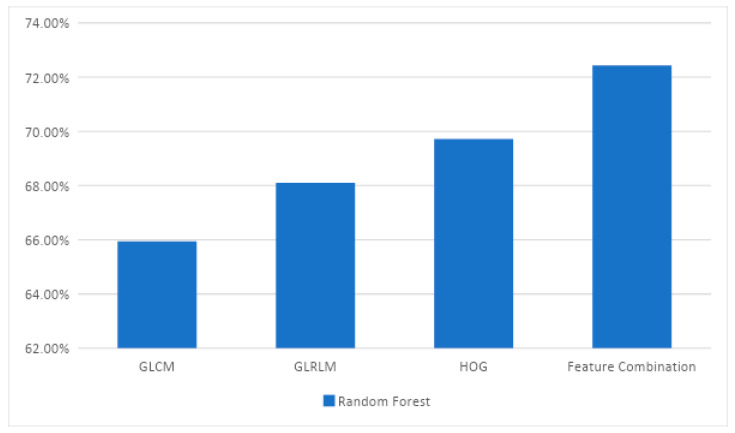
Comparison of accuracies by the random forest classifier for all feature extraction techniques on Dataset-I.

Figure 3 summarizes the accuracies of the random forest classifier with different feature extraction methods. Feature combination has obtained the highest accuracy of 72% among all other methods. Feature combination has resulted in improved accuracy than individual feature extraction methods.

#### 5.3.2. Dataset-II Results

Dataset-II contains cervigrams of only the aceto-whitening effect. As Dataset-I, similar experiments are performed on Dataset-II. The following subsections describe these experimental results.

- *GLRLM Feature Extraction for Dataset-II*

Table 10 summarizes the performance values of different classifiers by using the GLRLM feature extraction method. The random forest classifier has given the highest accuracy of 85.53% compared to other classifiers. This accuracy is higher than that of GLRLM of Dataset-I. This is because aceto-whitening images are more clearly distinguishable.

b. *GLCM Feature Extraction for Dataset-II*

Table 11 summarizes the experimentation results of different machine learning algorithms trained on GLCM features. The random forest has given the commendable accuracy of 77.37% compared to other classifiers. This accuracy is lower than the GLRLM feature extraction method.

c. *HOG Feature Extraction for Dataset-II*

Table 12 depicts the performance values of classifiers trained on HOG features. The random forest has given the better accuracy of 65.29% compared to other classifiers. The performance of HOG is lesser than that of GLRLM and GLCM methods. For aceto-whitening images, GLRLM has given the highest accuracy than HOG and GLCM.

d. *Hybrid Feature Fusion for Dataset-II*

Table 13 gives the performance values of different machine learning classifiers based on hybrid feature fusion vectors. Here, again, the random forest has given a good accuracy of 84.47% compared with other machine learning classifiers. This is the highest accuracy compared to individual feature extraction methods.

Table 14 shows the comparison of proposed method with existing studies in literature. It can be analyzed that the proposed method has given better accuracy than the existing methods.

## 6. Conclusions

This paper has presented the hybrid model ColpoClassifier, which consists of two phases, including feature extraction and classification of cervigrams. The formation of two datasets, namely Dataset-I and Dataset-II, is also a contribution of this paper. In ColpoClassifer, different feature extraction techniques, namely GLRLM, GLCM, and HOG, have been experimented, and the hybrid feature fusion is presented. These features are passed to different classifiers, and performance evaluation is conducted. From experiments, it is observed that, among individual classifiers, HOG has given the best accuracy of 69.72% for Dataset-I, and GLRLM has given the best accuracy of 85.53% for Dataset-II. The accuracies of hybrid feature fusion are 72.43% and 84.47% for Dataset-I and Dataset-II, respectively. These higher accuracies are given by the random forest classifier. In Summary, the hybrid feature fusion, along with the random forest classifier, has given a better performance than the others. It has also been observed that the usage of aceto-whitening images for cervical cancer diagnosis is important and gives the best performance. Because of the limited size of datasets, this paper has presented the experimental model based on handcrafted feature extraction methods. However, after having a large number of datasets, in future works, deep learning models can be customized for better performance.

## Figures and Tables

**Table 1:** GLRLM features.

Long-run emphasis	$LRE=\sum_{i=1}^{N_{g}}\;\sum_{j=1}^{N_{r}}P\left(i,j|\;\theta \right)/j^{2}/N_{r}\left(\theta \right)$
Short-run emphasis	$SRE=\sum_{i=1}^{N_{g}}\;\sum_{j=1}^{N_{r}}\frac{P_{\left(i,j|\;\theta \right)\;\;\;\;}}{j^{2}}$/$N_{r}$(θ)
Gray-level nonuniformity	$GLN=\sum_{i=1}^{N_{g}}\;{\left(\sum_{j=1}^{N_{r}}P_{\left(i,j|\;\theta \right)\;\;\;\;}\right)}^{2}/\;\;N_{r}\left(\theta \right)$
Run length nonuniformity	$RLN=\sum_{j=1}^{N_{r}}\;\;{\left(\sum_{i=1}^{N_{g}}P_{\left(i,j|\;\theta \right)\;\;\;\;}\right)}^{2}\;\;/N_{r}\left(\theta \right)\;\;$
Run percentage	$RP=N_{r}\left(\theta \right)/N_{p}$
Short-run low-Gray-level emphasis	$SRLGLE=\sum_{i=1}^{N_{g}}\;\sum_{j=1}^{N_{r}}\;\frac{P_{(i,j\;|\;\theta )\;\;}}{i^{2}j^{2}}/N_{r}\left(\theta \right)$
Long-run low-Gray-level emphasis	$LRLGLE=\sum_{i=1}^{N_{g}}\;\sum_{j=1}^{N_{r}}\;\frac{P_{(i,j\;|\;\theta )\;}j^{2}}{i^{2}}/N_{r}\left(\theta \right)$
Long-run high-Gray-level emphasis	$LRHLGLE=\sum_{i=1}^{N_{g}}\;\sum_{j=1}^{N_{r}}\;P_{(i,j\;|\;\theta )\;}i^{2}j^{2}/\;N_{r}\left(\theta \right)$

**Table 2:** Harlick features of GLCM.

Maximum Probability	Strongest Response of P, in the Range [0, 1] Max P_i,j_ I,j
Correlation	Calculates correlation between pixel I and neighboring pixel j in the range [0, 1] $\;\;\;\;\;\;\;\;\sum_{i=1}^{N_{g}}\;\sum_{j=1}^{N_{g}}\;\frac{P\left(i,j\right)ij-{µ}_{x}{µ}_{y}}{\sigma_{x}\left(i\right)\sigma_{y}\left(j\right)}$
Contrast	Calculates intensities between pixels in range [0, 1] $\sum_{i=1}^{N_{g}}\;\sum_{j=1}^{N_{g}}\;{\left(i-j\right)}^{2\;}P_{\left(i,j\right)}$
Energy	Energy will be 1 for constant image $\sum_{i=1}^{N_{g}}\;\sum_{j=1}^{N_{g}}\;P^{2}\;\left(i,j\right)$
Homogeneity	Calculates spatial auto-correlation for range [0, 1] $\sum_{i=1}^{N_{g}}\;\sum_{j=1}^{N_{g}}\;\frac{P_{\left(i,j\right)}}{1+\left|i-j\right|\;}$
Entropy	Calculates the randomness of the matrix$-\sum_{i=1}^{N_{g}}\;\sum_{j=1}^{N_{g}}P_{\left(i,j\right)}\;logP_{\left(i,j\right)}\;$

**Table 3:** Data augmentation.

Operation	Parameter Values
Rotation	15 degrees
Width Shift	0.2
Shear	0.2
Height Shift	0.2
Horizontal Flip	True
Vertical Flip	True
Fill Mode	Constant

**Table 4:** Dataset-I details.

Class	#
Abnormal images	229
Normal images	141
Total images	370

**Table 5:** Dataset-II details.

Class	#
Abnormal images	214
Normal images	166
Total images	380

**Table 6:** GLRLM Results on Dataset-I.

Classification Algorithm	Accuracy	Sensitivity	Specificity	Precision	Recall	MAE	F1 Measure
Naïve Bayes	49.45%	0.49	0.36	0.63	0.5	0.5	0.56
Bayes Net	45.94%	0.45	0.35	0.65	0.45	0.48	0.53
Random Tree	61.62%	0.61	0.43	0.61	0.61	0.38	0.61
Random Forest	68.11%	0.68	0.40	0.67	0.68	0.40	0.67
Decision Table	61.89%	0.61	0.61	0.67	0.61	0.47	0.64
Logistic	61.35%	0.61	0.52	0.58	0.61	0.43	0.59

**Table 7:** GLCM results on Dataset-I.

Classification Algorithm	Accuracy	Sensitivity	Specificity	Precision	Recall	MAE	F1 Measure
Naïve Bayes	61.89%	0.61	0.34	0.66	0.61	0.39	0.63
Bayes Net	63.24%	0.63	0.38	0.64	0.63	0.43	0.63
Random Tree	62.70%	0.62	0.41	0.62	0.62	0.37	0.62
Random Forest	65.94%	0.65	0.41	0.65	0.65	0.39	0.65
Decision Table	59.45%	0.59	0.56	0.55	0.59	0.46	0.57
Logistic	59.45%	0.59	0.49	0.58	0.59	0.42	0.58

**Table 8:** HOG results on Dataset-I.

Classification Algorithm	Accuracy	Sensitivity	Specificity	Precision	Recall	MAE	F1 Measure
Naïve Bayes	64.86%	0.64	0.40	0.64	0.64	0.42	0.64
Bayes Net	63.51%	0.63	0.49	0.61	0.63	0.44	0.62
Random Tree	59.18%	0.59	0.45	0.59	0.59	0.40	0.59
Random Forest	69.72%	0.69	0.42	0.69	0.69	0.40	0.69
Decision Table	63.51%	0.63	0.49	0.61	0.63	0.44	0.62
Logistic	61.89%	0.61	0.47	0.60	0.61	0.42	0.60

**Table 9:** Hybrid Feature Fusion on Dataset-I.

Classification Algorithm	Accuracy	Sensitivity	Specificity	Precision	Recall	MAE	F1 Measure
Naïve Bayes	55.40%	0.55	0.32	0.68	0.55	0.43	0.61
Bayes Net	53.51%	0.53	0.36	0.63	0.53	0.45	0.58
Random Tree	65.40%	0.65	0.39	0.65	0.65	0.34	0.65
Random Forest	72.43%	0.72	0.37	0.72	0.72	0.40	0.72
Decision Table	61.89%	0.61	0.52	0.59	0.61	0.44	0.60
Logistic	62.97%	0.63	0.41	0.63	0.63	0.38	0.63

**Table 10:** GLRLM results on Dataset-II.

Classification Algorithm	Accuracy	Sensitivity	Specificity	Precision	Recall	MAE	F1 Measure
Bayes Net	63.68%	0.63	0.45	0.67	0.63	0.36	0.65
Naïve Bayes	57.36%	0.57	0.37	0.63	0.57	0.43	0.60
Random Tree	78.15%	0.78	0.22	0.782	0.78	0.21	0.78
Random Forest	85.52%	0.85	0.16	0.856	0.85	0.26	0.85
Decision Table	68.15%	0.68	0.30	0.693	0.64	0.38	0.67
Logistic	80%	0.80	0.21	0.800	0.80	0.28	0.80

**Table 11:** GLCM results on Dataset-II.

Classification Algorithm	Accuracy	Sensitivity	Specificity	Precision	Recall	MAE	F1 Measure
Bayes Net	69.21%	0.69	0.30	0.69	0.69	0.31	0.69
Naïve Bayes	70.26%	0.70	0.28	0.71	0.70	0.30	0.70
Random Tree	69.21%	0.69	0.31	0.69	0.69	0.30	0.69
Random Forest	77.36%	0.77	0.24	0.77	0.77	0.30	0.77
Decision Table	72.63%	0.72	0.31	0.72	0.72	0.37	0.72
Logistic	74.47%	0.74	0.27	0.74	0.74	0.31	0.74

**Table 12:** HOG results on Dataset-II.

Classification Algorithm	Accuracy	Sensitivity	Specificity	Precision	Recall	MAE	F1 Measure
Bayes Net	63.68%	0.63	0.45	0.67	0.63	0.36	0.65
Naïve Bayes	56.29%	0.56	0.47	0.55	0.56	0.46	0.55
Random Tree	58.61%	0.58	0.43	0.58	0.58	0.41	0.58
Random Forest	65.29%	0.65	0.40	0.65	0.65	0.44	0.65
Decision Table	62.21%	0.62	0.46	0.62	0.62	0.46	0.62
Logistic	61.18%	0.61	0.42	0.60	0.61	0.45	0.60

**Table 13:** Hybrid feature fusion results for Dataset-II.

Classification Algorithm	Accuracy	Sensitivity	Specificity	Precision	Recall	MAE	F1 Measure
Bayes Net	75.00%	0.75	0.28	0.74	0.75	0.26	0.74
Naïve Bayes	71.31%	0.71	0.26	0.26	0.71	0.29	0.38
Random Tree	74.47%	0.74	0.27	0.74	0.74	0.25	0.74
Random Forest	84.47%	0.84	0.18	0.84	0.84	0.29	0.84
Decision Table	75.00%	0.75	0.29	0.74	0.75	0.34	0.74
Logistic	79.47%	0.79	0.21	0.79	0.79	0.21	0.79

**Table 14:** Comparison between the proposed method with the existing studies.

Approach	Accuracy
Deep Learning (Resnet-50) [14]	80.6%
ERSTCM + FL-HKSVM [20]	82.07%
Ensemble Deep Learning Structure [21]	84%
KELM [17]	79.98%
Proposed Model (Hybrid Feature Fusion) GLRLM + GLCM + HOG Dataset-II	84.47%

## Data Availability

Data will be available on request to authors.
